# Successful implementation of online educational lectures of the German Society for Radiation Oncology (DEGRO)

**DOI:** 10.1007/s00066-023-02162-x

**Published:** 2023-10-27

**Authors:** Marcel Büttner, Philip Melton, Rainer Fietkau, Cordula Petersen, Mechthild Krause, Kerstin Borgmann, Ulrich Wolf, Maximilian Niyazi, Hans Christiansen, Ulrike Höller, Daniela Schmitt, Lukas Käsmann, Philipp Linde, Daniel F. Fleischmann, Sonia Ziegler, Angelique Bresch, Matthias Mäurer

**Affiliations:** 1 Department of Radiation Oncology, University Hospital, LMU Munich, Munich, Germany; 2 LMU Munich, University Hospital, Munich, Germany; 3 Radiation Clinic, Erlangen University Hospital, Erlangen, Germany; 4 Department of Radiotherapy and Radiation Oncology, University Medical Center Hamburg-Eppendorf, Hamburg, Germany; 5 Department of Radiotherapy and Radiation Oncology and National Center for Radiation Research in Oncology (OncoRay), University Hospital Carl Gustav Carus, Medical Faculty, Technische Universität Dresden, Dresden, Germany; 6 partner site Munich, German Cancer Consortium (DKTK), Munich, Germany; 7 German Cancer Research Center (DKFZ), Heidelberg, Germany; 8 Department of Radiation Oncology, University Hospital Leipzig, Leipzig, Germany; 9 Clinic for Radiotherapy and Special Oncology, Hanover Medical School, Hanover, Germany; 10 MVZ Charité Vivantes, Berlin, Germany; 11 Department of Radiation Oncology, University Medical Center Göttingen, Göttingen, Germany; 12 Department of Radiation Oncology, Cyberknife and Radiation Therapy, Faculty of Medicine and University Hospital of Cologne, University of Cologne, Cologne, Germany; 13 Center for Integrated Oncology (CIO), University Hospital of Cologne, Faculty of Medicine and University of Cologne, Kerpener St 62, 50937 Cologne, Germany; 14 Office of the German Society for Radiation Oncology (DEGRO), Berlin, Germany; 15 Department for Radiotherapy and Radiation Oncology, University Hospital Jena, Friedrich-Schiller-University, Bachstr. 18, 07743 Jena, Germany; 16 Clinician Scientist Program “OrganAge”, Jena University Hospital, 07747 Jena, Germany; 17 partner site Dresden, German Cancer Consortium, Dresden, Germany; 18 partner site Dresden, National Center for Tumor Diseases, Dresden, Germany; 19 Helmholtz-Zentrum Dresden—Rossendorf, Dresden, Germany

**Keywords:** Medical education, Radiation oncology, Teaching format, Online webinar, e‑learning

## Abstract

**Purpose:**

Modern digital teaching formats have become increasingly important in recent years, in part due to the COVID-19 pandemic. In January 2021, an online-based webinar series was established by the German Society for Radiation Oncology (DEGRO) and the young DEGRO (yDEGRO) working group. In the monthly 120-minute courses, selected lecturers teach curricular content as preparation for the board certification exam for radiation oncology.

**Methods:**

The evaluation of the 24 courses between 01.2021 and 12.2022 was performed using a standardized questionnaire with 21 items (recording epidemiological characteristics of the participants, didactic quality, content quality). A Likert scale (1–4) was used in combination with binary and open-ended questions.

**Results:**

A combined total of 4200 individuals (1952 in 2021 and 2248 in 2022) registered for the courses, and out of those, 934 participants (455 in 2021 and 479 in 2022) later provided evaluations for the respective courses (36% residents, 35% specialists, 21% medical technicians for radiology [MTR], 8% medical physics experts [MPE]). After 2 years, 74% of the DEGRO Academy curriculum topics were covered by the monthly webinars. The overall rating by participants was positive (mean 2021: 1.33 and 2022: 1.25) and exceeded the curriculum offered at each site for 70% of participants. Case-based learning was identified as a particularly well-rated method.

**Conclusion:**

The DEGRO webinar expands the digital teaching opportunities in radiation oncology. The consistently high number of participants confirms the need for high-quality teaching and underlines the advantages of e‑learning methods. Optimization opportunities were identified through reevaluation of feedback from course participants. In its design as a teaching format for a multiprofessional audience, the webinar series could be used as a practice model of online teaching for other disciplines.

**Supplementary Information:**

The online version of this article (10.1007/s00066-023-02162-x) contains supplementary material, which is available to authorized users.

## Introduction

The complexity of radiation oncology has increased significantly in recent decades due to developments in both clinical and computer technologies [1, 2]. Additionally, increasing quality control requirements and rising patient numbers are making more structured and time-effective teaching across multiple sites a necessity [3]. To ensure the feasibility of these structural changes, prospective physicians must be able to develop the necessary knowledge, skills, and behaviors during their residency training [4–6].

To realize this status and to ensure broader and more standardized teaching, the European Society for Radiotherapy and Oncology (ESTRO) and the Academy of the German Society for Radiation Oncology (DEGRO Academy) have put forward several curricula for the training of radiation oncologists [7–9]. One example of the attempt to introduce standardization to the training of radiation oncologists is a complimentary module created by the ESTRO on clinical oncology to be used in conjunction with their core curriculum [10]. However, the examples of various entities continue to demonstrate that specific radiation oncology topics need to be instructed in a clear and structured manner for broader knowledge transfer [11–13].

A relatively new education model of instruction in radiation oncology is offered by the advancement of digitalization [14]. The development of information technology (IT) combined with the onset of the COVID-19 pandemic has led to the expansion of new digital teaching and learning methods [15, 16], some of which are currently also being applied to residency training [17]. Examples of existing implementations are e‑learning, online tumor boards, and telehealth (which includes education and health promotion), as well as telemedicine and teleconsultation [18]. Even after the pandemic, these advanced formats of digital teaching tools should be maintained [19].

The field of radiation oncology is rapidly evolving. To counterbalance the development of new radiation technologies, the clinical practice and associated skills of physicians must also be adapted [20]. New teaching formats offer a potential solution to this increasing challenge and could serve to improve the quality of clinical care by broadening the horizon of the respective physicians [21, 22]. To achieve this goal, these new formats must include practical, competency-based, and interdisciplinary approaches [5]. First of all, hands-on seminars are particularly important to ensure the smooth transition of theory into practice in radiation oncology [23]. Several approaches to improve practice-based teaching have already been successfully implemented in Canada, with innovative teaching methods, such as case libraries, computer-assisted learning, and quality assessments, revealing a very high degree of effectiveness [24]. Preliminary work carried out in Germany and other countries has identified and later concluded that the synergistic collaboration of multiple disciplines in the case-based teaching of radiation oncology is very important for the successful education of future residents [25–27]. Additionally, a study from Canada shows that case-based learning in anatomy and radiology contouring is an ideal candidate for transfer to online formats, and course participants particularly appreciate the flexible, self-paced learning environment [28].

Studies of other medical specialties have shown that online teaching formats are challenging due to lower completion rates but can, however, offer a very effective learning experience and other benefits such as geographic flexibility [28–30]. Within radiation oncology, online courses in the field have shown high effectiveness in delivering knowledge to both residents and students [2, 14, 31, 32]. Currently, there are already a number of independent online educational resources available to radiation therapists [33], and with the introduction of the DEGRO webinars there is now a first, continuous educational program with future relevance for this field. ESTRO has already achieved a high level of satisfaction with their pilot systematic gynecological online courses [34] and the evaluations of e‑learning courses indicate that the core competencies of radiation oncologists and other medical staff can be significantly increased [35]. However, the quality of teaching as well as a clear and concise structure of learning content are crucial to the success of digital education in radiation oncology [2]. The future lies in the integration of e‑learning in the form of learning videos and practical seminars [16]. The DEGRO webinars, which are the subject of this paper, were launched in January of 2021 and take place monthly with the aim of providing trainee physicians with an additional opportunity for continuing of education.

Our study aims to analyze the first results of the DEGRO webinar evaluations and draw conclusions on their ability to meaningfully contribute to the training of radiation oncologists in Germany and, moreover, German-speaking countries in Europe.

## Methods

The webinar program and the standardized questionnaire used for evaluation in this study were developed by representatives of the DEGRO Academy, the young DEGRO, the DEGRO Board, and representatives of medical physics and radiation Biology. The goal was to establish an educational program covering the entire curriculum of the DEGRO Academy within 3 years. The target groups were residents and specialists in radiation oncology as well as medical physics experts and technologists for radiology (MTR).

The questionnaire consisted of 21 items, with six questions using a Likert scale ranging from 1 to 4 (with 1 being the highest and 4 the lowest possible score) and one question on each course topic with a Likert scale ranging from 1 to 6 (with 1 being the highest and 6 the lowest possible score). An additional ten binary (yes/no) or multiple-choice questions and four optional open-ended questions were included to allow broader feedback. The selected items covered the perceived quality of the course, the degree of coverage of topics from the DEGRO curriculum (as assessed by the respective lecturer), and key epidemiological metrics of the participant’s evaluation of the respective course (see supplemental 1 for the full questionnaire) [9]. Course participants were asked to rate the course at the end of each session using the UmfrageOnline platform (created by enuvo GmbH, Switzerland). Only questionnaires in which the epidemiological parameters had been answered were included in this analysis.

The analysis of curriculum coverage was based on the DEGRO Academy curriculum version from 2018–2022 [9]. The assignment of the online courses to the curriculum was performed separately for each course by the respective lecturers in conjunction with the DEGRO Academy (100% indicates the coverage of all subtopics). The lecturers evaluated whether the respective subtopic of the curriculum in the online course represents a learning objective of the lecture in consultation with the DEGRO Academy.

All data were prepared using Microsoft Excel (Mac Version 16.64, Microsoft, Redmond, WA, USA) and statistically analyzed with GraphPad Prism (V9, GraphPad Software, San Diego, CA, USA).

## Results

A total of 4200 (1952 in 2021 and 2248 in 2022) participants enrolled in the courses and 934 (455 in 2021 and 479 in 2022) subsequently evaluated the respective course. This represents a response rate of approximately 22%. When registering for the online educational format, 905 (22%) of participants voluntarily indicated a medical practice or clinic in Germany (88%), Austria (10%), Switzerland (2%), Italy (> 1%), or Belgium (> 1%; see supplemental 2). Of the 934 course participants who rated their seminar, 67.0% were female. It is also noteworthy that the largest group of participants were doctors in specialist training (37%), while specialists were the second most represented group (35%). Clustering of the age distribution showed that the 25 to 40 age group made up the majority (51%). This can be reconciled with trends in years of training for physicians. There is a high percentage of participants who were older than 45 (41%). This percentage of 45-year-old participants increased between 2021 and 2022 (see Table 1 for further and more detailed information on the age distribution).

**Table 1:** Number of participants evaluated in 2021 and 2022 referring to the range of age distribution and their current occupation

	2021	2022	Total	
*No. of correctly submitted evaluations*	455	479	934	
*Sex (% of female participants)*	66.2%	67.8%	67.0%	
*Age distribution (no. of participants and % of total)*				
< 25 years old	4	3	7	(1%)
25–30 years old	68	55	123	(13%)
31–35 years old	107	86	193	(21%)
36–40 years old	83	77	160	(17%)
41–45 years old	25	47	72	(8%)
> 45 years old	168	211	379	(41%)
*Professional background (no. of participants)*				
Physician (first year of residency)	23	8	31	(3%)
Physician (second year of residency)	30	36	66	(7%)
Physician (third year of residency)	23	31	54	(6%)
Physician (fourth year of residency)	34	33	67	(7%)
Physician (fifth year of residency)	44	45	89	(10%)
Physician (sixth year or more of residency)	19	22	41	(4%)
Specialist	167	159	326	(35%)
Medical physicist in training	4	2	6	(1%)
Medical physicist	28	35	63	(7%)
Radiation biologist	1	0	1	(0%)
Medical technical radiation assistant	70	93	163	(17%)
Radiation technician	12	15	27	(3%)

**Fig. 1:**
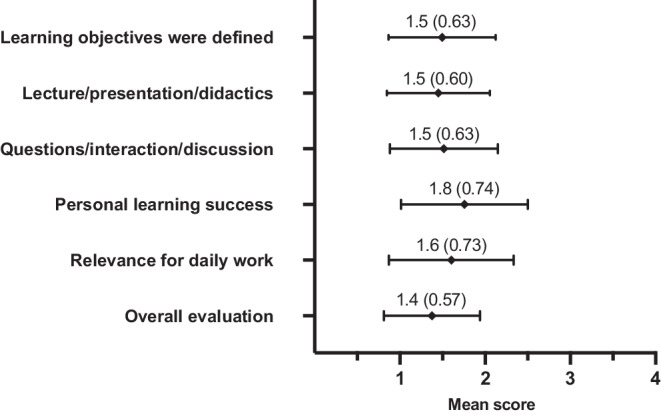
The graph shows the mean score (and standard deviation) across 2021 and 2022 of the course participants’ evaluations for individual aspects of the respective course and the total course evaluation on a Likert scale

In order to determine overall course satisfaction and to identify potential issues and areas for improvement, the questionnaire contained numerous items pertaining to both the content and didactic quality of the online seminars. Figure 1 shows the course participants’ responses to the questionnaire items using a Likert scale. These items were included to track the development of course quality with a quantitative readout. Key findings were primarily that mean scores never dropped below 2 in any category and either remained constant or improved between 2021 and 2022 (see supplemental 3).

**Fig. 2:**
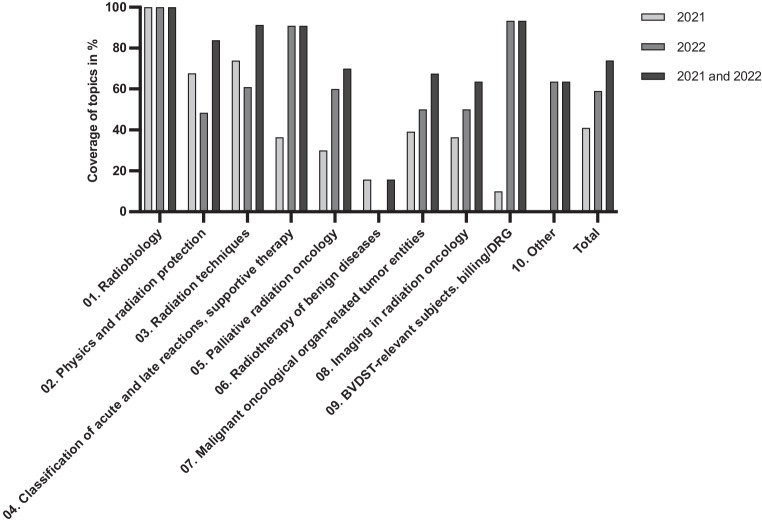
Percentage of the ten subjects contained within the DEGRO curriculum required for specialization in radiation oncology that were covered by the online seminars in 2021 and 2022 (topic 09 BVDST (*Bundesverband Deutscher Strahlentherapeuten*) includes health economic and financial topics)

Further results showed the courses regarding the thematic coverage of the DEGRO curriculum. In Germany, DEGRO recommends that specialist candidates are instructed in ten different areas in order to obtain their final degree [9]. To this end, we analyzed the percentage of this curriculum covered by our online seminars (Fig. 2 and supplemental 4). Overall, 74% of the DEGRO curriculum was covered by online seminars in 2021 and 2022. Except for topic 6, all subjects were covered at least 60% of the time in 2021 and 2022, with topics 1–4 and 9 covered more than 80% of the time. However, topic 6 (radiotherapy of benign diseases) was covered only 15.6% of the time in 2021 and 0% in 2022.

**Fig. 3:**
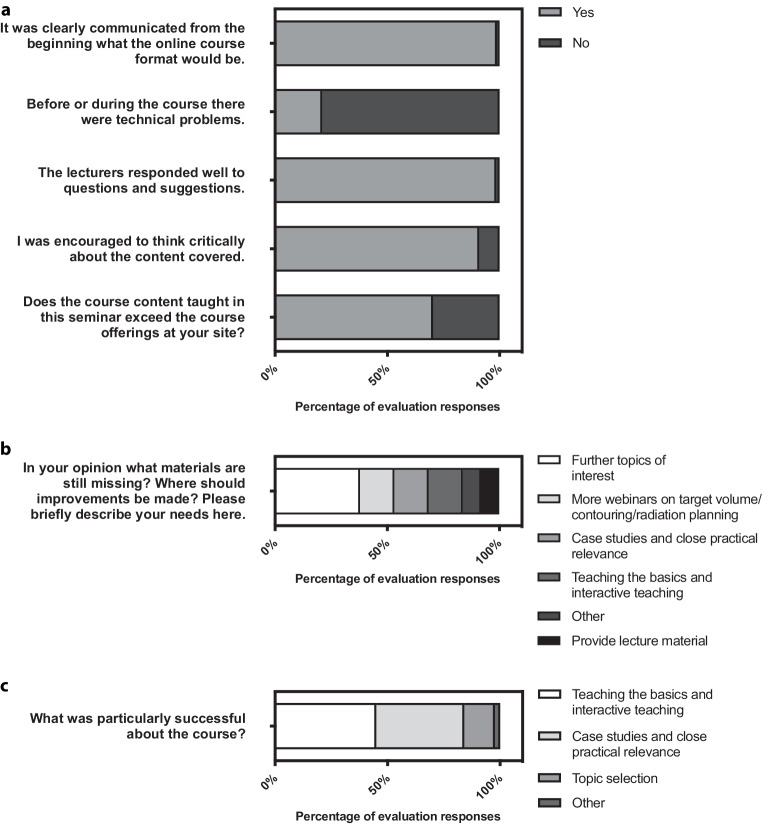
**a** Responses to the binary questions contained in the questionnaire. **b** Optional open question, 111 responses were submitted. **c** Optional open question, 169 responses were submitted

The diagrams in Fig. 3 depict the results from the binary and open questions on course quality. For 70% of participants, the online courses outperform the course offerings at their site. Additionally, 91% of submitted evaluations responded with “yes” to the question of whether they were encouraged to think critically about the course content. However, there were also areas for improvement identified. For instance, nearly 21% of course participants reported that they had encountered technical difficulties during their course attendance.

In addition, the open-ended question in Fig. 3b shows that the most important areas of perceived improvement potential among the 111 responses were as follows (in descending order): more topics of interest (37.8%); more webinars on target volume/contouring/radiation planning (15.3%); case studies and close practical relevance (15.3%); teaching fundamentals and interactive teaching (15.3%). The perceived positive aspects of the courses were distributed as shown in Fig. 3c, with the two most frequent remarks being teaching the basics and interactive teaching (45.0%); case studies and close practical relevance (39.1%).

## Discussion

Consistent structuring and standardization of radiotherapy education, especially considering current and future concerns, is an inevitable necessity for maintaining internationally competitive and high-quality teaching [6, 7, 36–38]. The DEGRO webinar established in 2021 makes an important contribution to meeting the need for practice-oriented education and training [3, 39].

Webinar participants believe that curricular learning objectives are defined within the webinars and provide a steep learning curve for daily work (Fig. 1). Given the high relevance of critical thinking to the quality of medical care [40], almost all participants (91%) reported having been trained in this area through the webinars (Fig. 3a). These results demonstrate the added value of curriculum-based training and indicate the potential for improvement in the quality and standardization of radiotherapy care in Germany. The participation rate of approximately 35% specialists shows that the courses continue to be visited after the board certification exam for radiation oncology. This is possibly due to the dynamic environment of the content in the field of oncology and that specialists also require courses to maintain their knowledge of current treatment guidelines. Possibly against the background of a lack of time resources for personal continuing education (journal clubs, congress attendance in presence), the specialists particularly appreciate the condensed and clearly presented knowledge transfer by proven experts in the respective subject area.

In addition to implementation of broad standardized teaching of the topics of the DEGRO curriculum, the didactic quality of the webinars was also the focus of our analysis. The course participants rated the current emphasis on the professional and practical relevance of the courses with the help of clinical cases as particularly successful (Fig. 3c), which, as described in preliminary work, show a very high teaching effectiveness [5, 20, 23]. In addition, 15% of the responses of participants showed an interest in the further expansion of case studies and close practical relevance (Fig. 3b) and a large proportion wished for further webinars on “target volume/contouring/radiation planning.” These results indicate the need for practice-oriented case-based online courses on target volume/contouring/radiation planning, which have previously been demonstrated to be effective [24, 26]. Another finding is that the categories “teaching the basics and interactive teaching” and “case studies and close practical relevance” were seen as both strengths and areas for improvement by the participants. This shows that the course already represents a desired format, but that this can be further developed.

The overall rating of approximately 1.4 (Fig. 1) indicates a good reception of the courses. In combination with the geographically flexible accessibility of e‑learning, the courses offer enormous potential for uniform location-independent high-quality teaching—also including German-speaking European colleagues in countries such as Austria or Switzerland (see supplemental 2).

For more than 70% of the participants, the course content goes beyond that offered at the respective site (Fig. 3a). This becomes particularly important when considering that the availability of broad teaching is a problem at smaller medical sites [41, 42]. This observation raises the question of a general teaching deficit and its causes. It underlines the importance of diverse and accessible teaching offers. In addition to location-independent teaching, networking between locations offers further potential. Even if participants only get to know each other online, this educational platform could lead to positive results such as cross-location collaborations. Figures 1 and 3a show that the webinars added significant value to the teaching offered in 2021 and 2022. However, at the same time, over these 2 years of the COVID-19 pandemic, there were significant limitations in the teaching offered at the sites as compared to previous years. Therefore, the effect of these evaluations can only be projected to future years to a limited extent and further evaluations in the coming years remain important. In addition, the concept of webinars allows for less direct interaction or individualized instruction. These tasks remain with teaching at the respective site, which continues to emphasize its importance. This highlights the possibility of the DEGRO seminars becoming an additional pillar of radiation oncology specialist training in the future.

Within 2 years alone, 74% of the DEGRO curriculum [9] was covered, and only one of ten major topics was covered at less than 60% of the time (Fig. 2). A physician in advanced training for radiation therapy and radiation oncology requires approximately 5 years to receive their degree according to German medical training regulations 2018 of the German Medical Association [43]. Extrapolated, all topics of the curriculum are covered within 5 years. While for 5 out of 10 main oncological topics 80% of the learning material was already covered by the webinars, there is potential for improvement for the topic area “radiotherapy of benign diseases” (Fig. 2). The underrepresentation of benign diseases in the observation period of this interim evaluation is due to the fact that one course on this topic was cancelled. In addition, since the series of courses is organized over a total period of 3 years, certain topics are not taught until later, so that a temporal distortion can occur. At the end of the 3 years, full coverage of the curriculum is achieved. Nevertheless, when planning the program, the aim should be to have as diverse a subject arrangement as possible in order to be able to react flexibly to shortfalls and to regularly cover subareas. Other possible outlooks of this format are the expansion into European countries with comparable residency training and offering the courses in English. The development of an online media library with video recordings, practice material, and exemplary specialist examinations are further desirable extensions. Analysis of the free-text responses from participants indicates that more case examples and practical guidance regarding contouring, target volume definition, and dose prescription are desired for future events. Further optimization of the event series could be to include interdisciplinary topics involving experts from other oncology departments.

In summary, our results confirm that DEGRO webinars already have value for creating a more structured and standardized specialist training in Germany. They are a well-received addition to the existing on-site education offering with worldwide availability and use proven e‑learning methods. We are convinced that tailoring the webinars to feedback from course participants’ will lead to better results and that they can serve as an example for other medical subspecialties to improve their continuing educational programs.

## Conclusion

The DEGRO webinar expands the digital teaching offer in radiation oncology. The consistently large number of participants confirms the need for high-quality teaching and underscores the advantages of e‑learning methods. The format was evaluated with a mean score of 1.4 across 2021 and 2022. Optimization opportunities were identified by re-evaluating feedback from course participants. In its design as a teaching format for a multiprofessional audience, the webinar could serve as a model for other disciplines.

### Supplementary Information

In this supplementary information you will find the original questionnaire of the webinars from 2021 and 22 (Supp. 1).

In addition, you will find an overview with the participants in relation to their place of residence Supp 2).

Finally, we have compiled the further evaluation results from the years 2021 and 2022 (Supp. 3).

Supplemental 4 compiles the coverage of the DEGRO curriculum through the webinars.
