# Supplementary material for: Successful implementation of online educational lectures of the German Society for Radiation Oncology (DEGRO)
Source: Strahlenther Onkol. 2023 Oct 27;200(2):151–8. doi: 10.1007/s00066-023-02162-x (PMC10805975; doi:10.1007/s00066-023-02162-x)
Supplement: Supplementary file 1 — In this supplementary information you will find the original questionnaire of the webinars from 2021 and 22 (Supp. 1). [file 66_2023_2162_MOESM1_ESM.pdf]

DEGRO

Deutsche Gesellschaft für Radioonkologie e. V.
AG Junge DEGRO & DEGRO-Akademie

Evaluation questionnaire 2021 and 2022

Evaluation zum Webinar

Es wurde von Beginn an klar kommuniziert, wie das Online-Kursformat aussehen wird. *

- ◯ ja
- ◯ nein

Was hat in der Vorab-Kommunikation gefehlt?

Vor oder während der Lehrveranstaltung gab es technische Probleme. *

- ◯ ja
- ◯ nein

Falls ja, bitte beschreiben Sie kurz, welche technischen Probleme auftraten:

Wie bewerten Sie die folgenden Vortragsteile? *

	Sehr gut	gut	befriedigend	ausreichend	mangelhaft	nicht beurteilbar
Vortrag 1	◯	◯	◯	◯	◯	◯
Vortrag 2	◯	◯	◯	◯	◯	◯
Vortrag 3	◯	◯	◯	◯	◯	◯
Vortrag 4	◯	◯	◯	◯	◯	◯

Die Dozenten gingen gut auf Fragen und Anregungen ein. *

◯ ja

◯ nein

Ich wurde zur kritischen Auseinandersetzung mit den behandelten Inhalten angeregt. *

◯ ja

◯ nein

Zu welchen Inhalten fehlen Ihrer Ansicht nach noch Materialien? An welcher Stelle sollte noch nachgebessert werden?
Bitte schildern Sie hier kurz Ihren Bedarf:

Besonders gelungen fand ich an der Lehrveranstaltung:

Welche weiteren Ideen würden diese Seminarreihe noch bereichern?

- ◯ Mehr Fallbeispiele
- ◯ Mehr interaktive Diskussionen
- ◯ Mehr Lerneinheiten zur Medizinphysik
- ◯ Mehr Lerneinheiten zur Strahlentherapie
- ◯ Andere [ ]

Übersteigt der in diesem Seminar vermittelte Lernstoff das Lehrangebot an ihrem Standort?

- ◯ ja
- ◯ nein

Grundsätzliche Fragen zur Veranstaltung

Lernziele wurden definiert *

- ◯ Sehr gut
- ◯ Gut
- ◯ Befriedigend
- ◯ Ausreichend

Vortrag/Präsentation/Didaktik *

- ◯ Sehr gut
- ◯ Gut
- ◯ Befriedigend
- ◯ Ausreichend

Fragen/Interaktion/Diskussion *

- ◯ Sehr gut
- ◯ Gut
- ◯ Befriedigend
- ◯ Ausreichend

Persönlicher Lernerfolg *

- ◯ Sehr gut
- ◯ Gut
- ◯ Befriedigend
- ◯ Ausreichend

Relevanz für die tägliche Arbeit *

- ◯ Sehr gut
- ◯ Gut
- ◯ Befriedigend
- ◯ Ausreichend

Gesamtbeurteilung *

- ◯ Sehr gut
- ◯ Gut
- ◯ Befriedigend
- ◯ Ausreichend

Seite 3

Sie sind...

- Weiblich
- Männlich
- Keine Angabe

Zu welche Altersgruppe gehören Sie?

- bis 25 Jahre
- 25-30 Jahre
- 31-35 Jahre
- 36-40 Jahre
- 41-45 Jahre
- >45 Jahre

In welchem Ausbildungsstand befinden Sie sich?

- ◯ Arzt bzw. Ärztin in Weiterbildung (1.Jahr)
- ◯ Arzt bzw. Ärztin in Weiterbildung (2.Jahr)
- ◯ Arzt bzw. Ärztin in Weiterbildung (3.Jahr)
- ◯ Arzt bzw. Ärztin in Weiterbildung (4.Jahr)
- ◯ Arzt bzw. Ärztin in Weiterbildung (5.Jahr)
- ◯ Arzt bzw. Ärztin in Weiterbildung (≥6.Jahr)
- ◯ Facharzt/Fachärztin
- ◯ Medizinphysiker/in in Weiterbildung
- ◯ Medizinphysiker/Medizinphysikerin
- ◯ Strahlenbiologe/Strahlenbiologin
- ◯ MTRA
- ◯ Radiologietechnologin/Radiologietechnologe

Wie haben Sie von dem Webinar erfahren?

- ◯ DEGRO Mailverteiler
- ◯ jDEGRO Email-Verteiler
- ◯ Kolleginnen und Kollegen
- ◯ Andere ____________

Die Umfrage ist beendet. Vielen Dank für die Teilnahme.

Das Fenster kann nun geschlossen werden.

Deutsche Gesellschaft für Radioonkologie e. V.
AG Junge DEGRO & DEGRO-Akademie

Evaluation questionnaire 2021 and 2022 - english translation

Webinar evaluation

It was clearly communicated from the beginning what the online course format would be. *

◯ yes no

◯

What was missing in the advance communication?

[ ]

There were technical problems before or during the course. *

◯ yes

◯ no

If yes, please briefly describe what technical problems occurred:

[ ]

How would you rate the following parts of the presentation? *

	Very good	good	satisfactory	sufficient	deficient	Not assessable
Lecture 1	◯	◯	◯	◯	◯	◯
Lecture 2	◯	◯	◯	◯	◯	◯
Lecture 3	◯	◯	◯	◯	◯	◯
Lecture 4	◯	◯	◯	◯	◯	◯

The instructors responded well to questions and suggestions. *

◯ yes

◯ no

I was encouraged to critically examine the content covered. *

◯ yes

◯ no

In your opinion, what materials are still missing? Where should improvements be made?
Please briefly describe your needs here:

What I found particularly successful about the course was:

What other ideas would enhance this seminar series?

- ◯ More case studies
- ◯ More interactive discussions
- ◯ More learning units on medical physics
- ◯ More learning units on radiation therapy
- ◯ Other ______

Does the material taught in this seminar exceed the course offerings at their location?

- ◯ yes
- ◯ no

Basic questions about the event

Learning objectives have been defined *

- ◯ Very good
- ◯ Good
- ◯ Satisfactory
- ◯ Sufficient

Lecture/Presentation/Didactics *

- ◯ Very good
- ◯ Good
- ◯ Satisfactory
- ◯ Sufficient

Questions/interaction/discussion *

- ◯ Very good
- ◯ Good
- ◯ Satisfactory
- ◯ Sufficient

Personal learning success *

- ◯ Very good
- ◯ Good
- ◯ Satisfactory
- ◯ Sufficient

Relevance for daily work *

- ◯ Very good
- ◯ Good
- ◯ Satisfactory
- ◯ Sufficient

Overall assessment *

- ◯ Very good
- ◯ Good
- ◯ Satisfactory
- ◯ Sufficient

Page 3

You are...

- ◯ Female
- ◯ Male
- ◯ Not specified

What age group do you belong to?

- ◯ Until 25 years
- ◯ 25-30 years
- ◯ 31-35 years
- ◯ 36-40 years
- ◯ 41-45 years
- ◯ >45 years

What level of training are you in?

- ◯ Physician in further training (1st year)
- ◯ Physician in further training (2nd year)
- ◯ Physician in further education (3rd year)
- ◯ Physician in further training (4th year)
- ◯ Physician in further education (5th year)
- ◯ Physician in postgraduate training (≥6.year)
- ◯ Specialist
- ◯ Medical physicist in advanced training
- ◯ Medical physicist
- ◯ Radiation biologist
- ◯ MTRA
- ◯ Radiologic technologist

How did you hear about the webinar?

- ◯ DEGRO Mail Distribution List
- ◯ jDEGRO Email Distribution List
- ◯ Colleagues
- ◯ Other [ ]

The survey is closed. Thank you for your participation.

The window can now be closed.
