# Supplementary material for: Successful implementation of online educational lectures of the German Society for Radiation Oncology (DEGRO)
Source: Strahlenther Onkol. 2023 Oct 27;200(2):151–8. doi: 10.1007/s00066-023-02162-x (PMC10805975; doi:10.1007/s00066-023-02162-x)
Supplement: Supplementary file 2 — In addition, you will find an overview with the participants in relation to their place of residence Supp 2). [file 66_2023_2162_MOESM2_ESM.pdf]

**Supplemental 2** When registering for the webinars, 905 (22%) of participants voluntarily indicated a medical practice or clinic in 2021 and 2022. **A)** In Europe Germany (88%), Austria (10%), Switzerland (2%), Italy (> 1%), or Belgium (> 1%) are represented. **B)** The most represented states in Germany are North Rhine-Westphalia, Baden-Wuerttemberg, Bavaria, and Lower Saxony.

**A**

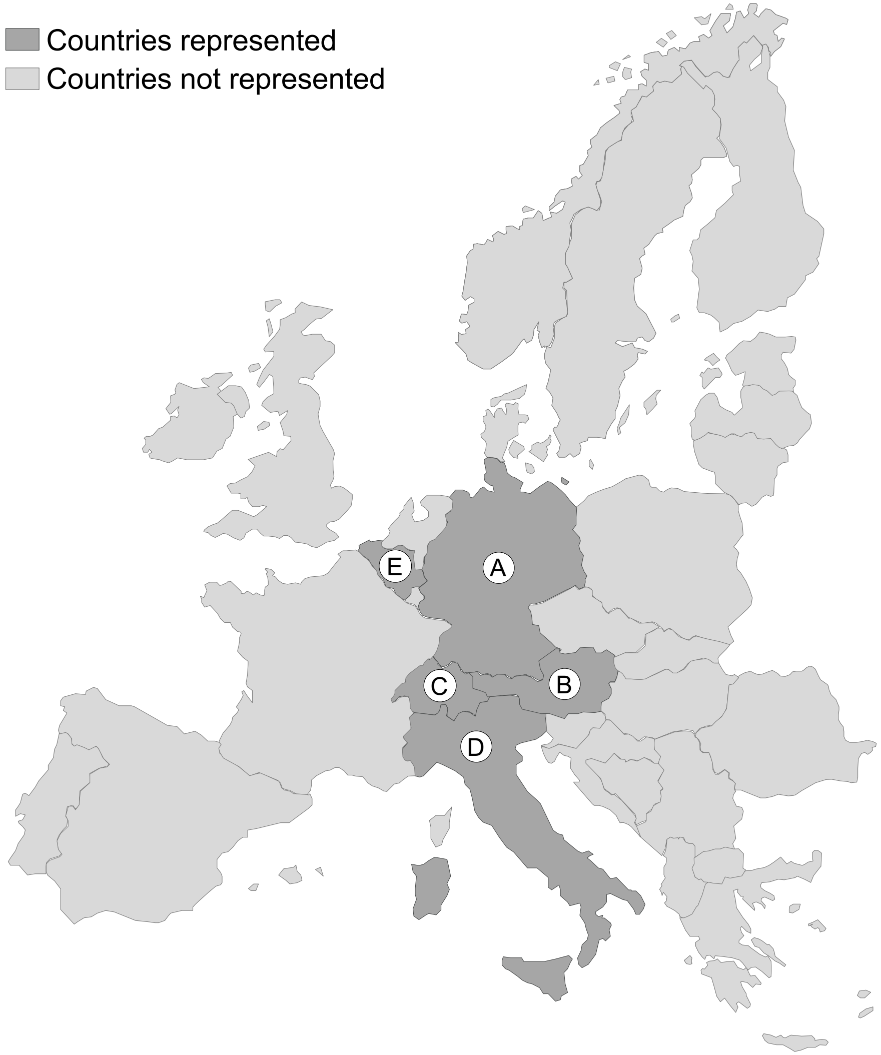


**B**

| | Location | 2021 | 2022 | 2021/ 2022 |
|---|---|---|---|---|
| **A** | **Germany** | **474** | **325** | **799** |
| 1. | North Rhine-Westphalia | 142 | 79 | 221 |
| 2. | Baden-Wuerttemberg | 73 | 47 | 120 |
| 3. | Bavaria | 57 | 45 | 102 |
| 4. | Lower Saxony | 40 | 16 | 56 |
| 5. | Hesse | 23 | 22 | 45 |
| 6. | Berlin | 25 | 17 | 42 |
| 7. | Saxony | 21 | 16 | 37 |
| 8. | Bremen | 19 | 17 | 36 |
| 9. | Thuringia | 10 | 17 | 27 |
| 10. | Hamburg | 10 | 10 | 20 |
| 11. | Schleswig-Holstein | 8 | 11 | 19 |
| 12. | Mecklenburg-Western Pomerania | 13 | 5 | 18 |
| 13. | Rhineland-Palatinate | 10 | 7 | 17 |
| 14. | Brandenburg | 8 | 8 | 16 |
| 15. | Saarland | 10 | 4 | 14 |
| 16. | Saxony-Anhalt | 5 | 4 | 9 |
| **B** | **Austria** | **50** | **36** | **86** |
| **C** | **Switzerland** | **8** | **9** | **17** |
| **D** | **Italy** | **2** | **0** | **2** |
| **E** | **Belgium** | **1** | **0** | **1** |
| **Total** | | **535** | **370** | **905** |
