# Supplementary material for: Successful implementation of online educational lectures of the German Society for Radiation Oncology (DEGRO)
Source: Strahlenther Onkol. 2023 Oct 27;200(2):151–8. doi: 10.1007/s00066-023-02162-x (PMC10805975; doi:10.1007/s00066-023-02162-x)
Supplement: Supplementary file 3 — Finally, we have compiled the further evaluation results from the years 2021 and 2022 (Supp. 3). [file 66_2023_2162_MOESM3_ESM.pdf]

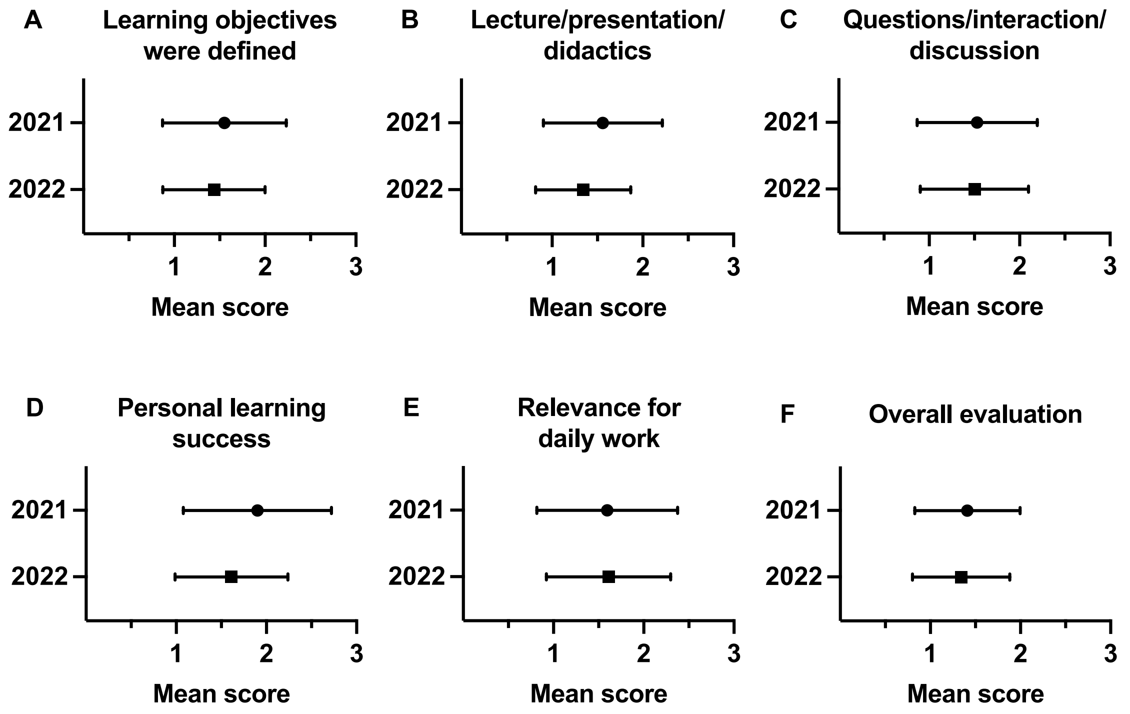

Supplement 3 The graph shows the mean score (μ) and standard deviation (SD) across 2021 and 2022 of the course participants` evaluations for individual aspects of the respective course and the total course evaluation on a Likert scale. **A)** 2021: μ=1.55; SD=0.68; 2022: μ=1.44; SD=0.56. **B)** 2021: μ=1.56; SD=0.66; 2022: μ=1.34; SD=0.52. **C)** 2021: μ=1.53; SD=0.66; 2022: μ=1.5; SD=0.6. **D)** 2021: μ=1.9; SD=0.82; 2022: μ=1.61; SD=0.63. **E)** 2021: μ=1.6; SD=0.78; 2022: μ=1.61; SD=0.69. **F)** 2021: μ=1.41; SD=0.58; 2022: μ=1.34; SD=0.54.
