# Supplementary material for: Successful implementation of online educational lectures of the German Society for Radiation Oncology (DEGRO)
Source: Strahlenther Onkol. 2023 Oct 27;200(2):151–8. doi: 10.1007/s00066-023-02162-x (PMC10805975; doi:10.1007/s00066-023-02162-x)
Supplement: Supplementary file 4 — Supplemental 4 compiles the coverage of the DEGRO curriculum through the webinars. [file 66_2023_2162_MOESM4_ESM.pdf]

Supplemental 4 Total number of subtopics of the 10 main topics and total of the DEGRO curriculum and number and percentage of subtopics covered by the webinars in 2021, 2022 and both years combined.

	Total number of subtopics in the Curriculum	#			%		
		2021	2022	2021/ 2022	2021	2022	2021/ 2022
01. Radiobiology	14	14	14	14	100.0%	100.0%	100.0%
02. Physics and radiation protection	31	21	15	26	67.7%	48.4%	83.9%
03. Radiation techniques	23	17	14	21	73.9%	60.9%	91.3%
04. Classification of acute and late reactions, supportive therapy	22	8	20	20	36.4%	90.9%	90.9%
05. Palliative radiation oncology	30	9	18	21	30.0%	60.0%	70.0%
06. Radiotherapy of benign diseases	19	3	0	3	15.8%	0.0%	15.8%
07. Malignant oncological organ-related tumor entities, incl. radiochemotherapy and targeted drugs	74	29	37	50	39.2%	50.0%	67.6%
08. Imaging in radiation oncology	22	8	11	14	36.4%	50.0%	63.6%
09. BVDST-relevant subjects, billing/DRG	30	3	28	28	10.0%	93.3%	93.3%
10. Other	11	0	7	7	0.0%	63.6%	63.6%
**Total**	**276**	**112**	**164**	**204**	**40.6%**	**59.4%**	**73.9%**
